# Effects of Priming on Problem Solving in Medical and Paramedical Students: A Study

**DOI:** 10.7759/cureus.36526

**Published:** 2023-03-22

**Authors:** Hari Om Vaja, Bhavan T Jikar, Khushboo Mathuria, Minakshi Parikh

**Affiliations:** 1 Psychiatry, B.J. (Byramjee Jeejeebhoy) Medical College, Ahmedabad, IND

**Keywords:** gender, paramedical students, medical students, problem solving, priming

## Abstract

This study aimed to evaluate the impact of priming on an individual's problem-solving ability, investigate the existence and direction of the relationship, and examine whether gender differences influence the extent of priming. A sample of 625 undergraduate medical and paramedical students was randomly assigned to either a positive or negative priming group. Data was collected through online forms using Ditloid puzzles, which assess a person's linguistic and word-forming (verbal) ability. The results showed a statistically significant difference between the positively primed group, which had a higher number of correctly answered questions, and the negatively primed group. Additionally, females outperformed males in the number of correctly answered questions, but the extent of priming was similar for both genders, with no significant difference between them.

## Introduction

In the words of Henry Ford, “Whether you think you can, or you think you can’t, you are right” [1], a concept that many famous and successful figures have propagated, a concept so simple in its being but with a rather immense effect on one’s life.

Research has indicated that individuals with a negative mindset towards a situation or problem tend to exhibit lower levels of creativity and persistence, becoming fatigued more easily. This limited mindset can hinder problem-solving efforts and lead to premature abandonment of the task at hand, often resulting in labelling the problem as "difficult". Conversely, those with a positive outlook tend to approach problems with an open mind, enabling them to consider a variety of perspectives and potential solutions. This expanded problem-solving approach can increase the likelihood of success [2].

It is evident that both negative and positive outlooks have their respective advantages and disadvantages. For instance, a negative outlook can be beneficial in a situation where speed is essential, such as when being chased by a dangerous animal. This type of narrow-minded focus is only achievable with a negative outlook. Conversely, a positive outlook can be beneficial when tackling day-to-day problems, as it allows for a greater range of possible solutions owing to a broadened attention and cognition, thus helping to find the best available solution [3]. As explained, both perspectives have their own use, but since we don’t face situations with fight-or-flight clauses in them commonly, we find that people with a positive outlook on a problem will find it easier to reach the answer.

In a study conducted in Turkey, it was sought to find the effect of a student’s belief in a problem-solving process. What they found was that the students with positive beliefs could persevere longer to find a solution to the given problem and found novel ways to do the same. In contrast, the ones with negative beliefs found it difficult to reach a solution. This study concluded that students’ beliefs influenced the decisions they made [2].

With the current study, we endeavour to find the effect of positive and negative priming on a person’s problem-solving ability. The methodology for this study intends to induce certain beliefs regarding the difficulty of the problem. Priming considers how a person’s judgement is affected when they do not versus do consciously associate the activation with the judgement they are making and do not versus do consciously intend to use the activated representation while forming that judgement [4]. Another study stated that the influence of priming on the subject occurred outside of either their (a) awareness of the possible effect of the prime or (b) intention to use the prime’s influence during judgment or action [5,6], i.e., the effects of the priming are assumed to occur due to either the absence of knowledge of the effects of the priming or the lack of intention to use such knowledge while responding to the questions. This is because if the person recognises the prime and utilises the said knowledge while solving the questions, the effects of the prime may disappear or even reverse [4,6].

To our knowledge, there has not been a study similar to the one stated above conducted in India. Therefore, we seek to conduct a study in order to answer the given question and determine the extent to which priming can influence the outcome of a situation, since this is an experience that many of us have encountered at some point in our lives.

## Materials and methods

This study hypothesises the effects of priming on the results of a task. Our null hypothesis is that no statistically significant difference exists between the results of the Easy (positively primed) and the Hard (negatively primed) groups. Our alternate hypothesis is to have a more positive outcome for those who are positively primed, and a more negative outcome for the negatively primed, i.e., we expect the ones in the Easy group to score more than the ones in the Hard group.

A cross-sectional observational study was conducted with approval from the Institutional Ethics Committee, B.J. Medical College & Civil Hospital, Ahmedabad, India (approval number: EC/Approval/15/2022/19/01/2022). A total of 625 participants were enrolled, 300 in the negatively-primed group (Hard group) and 325 in the positively-primed group (Easy group). The minimum number of participants required was decided based on the statistical formula available for sample-size calculation in a cross-sectional study. We conducted the study via an online questionnaire in medical and para-medical colleges of Gujarat, India. The inclusion criteria were adult medical or paramedical students who gave their consent via an online consent form. Adult medical or paramedical students who did not give their consent or individuals with any psychiatric illness were excluded.

To collect the data from the participants, Google Forms (Google LLC, Mountain View, California, United States) was used consisting of a consent form, personal details including name, gender, and history of psychiatric illness, and 10 Ditloid puzzles standardized by the Department of Psychiatry, B.J. Medical College, Ahmedabad.

The participants were instructed beforehand in the forms regarding the method to fill in the answers to the questions, followed by their consent to the same study. We asked each participant a series of 10 questions, which were the same for all, but every individual subject was primed differently, in accordance with the group they were randomly assigned to. We also repeatedly stated that the questions that the participants face are Hard/Easy in accordance with the group they are assigned to, to enhance the effect of priming [7,8]. 

Positively primed participants were provided instructions indicating that the question category will be "Easy," accompanied by a 1-to-10 difficulty scale. We further reinforced this perception by rating the difficulty of each individual question from 1 to 4 out of 10. Negatively primed participants were instructed that the category of questions they will face is "Hard," and a scale of 1-to-10 was provided in the instructions. Each individual question was rated 6 to 10 out of 10 to emphasize its difficulty. To reinforce the perception of difficulty, the form had a red theme, which had been associated with negative emotions and difficulty in previous research [9]. Participants were also informed that they may choose to leave the questions if they find them too difficult. Furthermore, in both questionnaires, we reinforced the credibility of the difficulty scale by specifying that the questions were rated based on a prior survey.

For the purpose of data analysis, we used IBM SPSS Statistics for Windows, Version 28.0 (Released 2021; IBM Corp., Armonk, New York, United States) and Microsoft Excel (Microsoft Corporation, Redmond, Washington, United States). Demographic characteristics were subjected to descriptive analysis, while unpaired t-tests were utilized to compare the mean scores between the Easy and the Hard groups, as well as between males and females.

## Results

**Figure 1:**
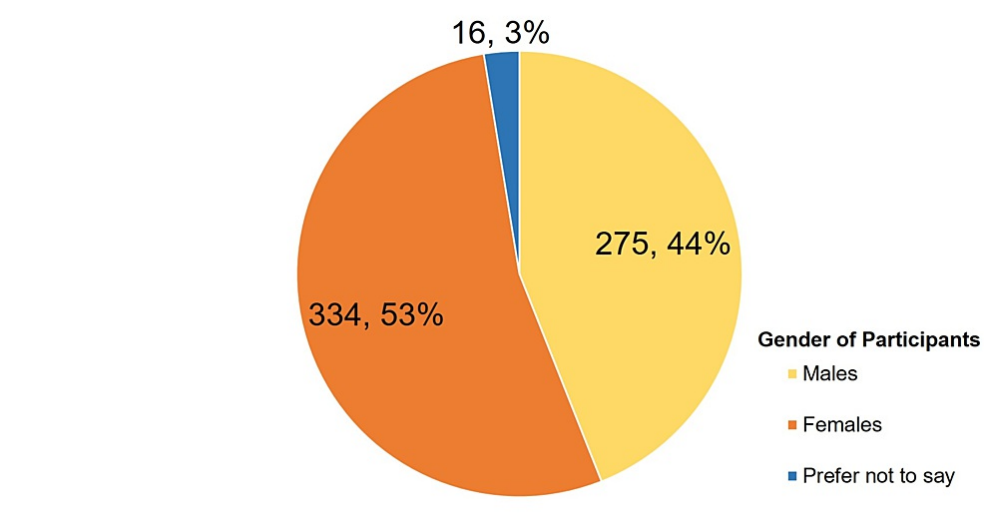
Gender-wise distribution of the participants (n, %)

A total of 625 persons participated in the study including 334 (53.44%) females, 275 (44%) males, and 16 (2.56%) who preferred not to reveal their gender. All the participants were matched to be in the age group of 18-25 years and were students of medical or paramedical colleges in Gujarat. The gender distribution of the participants has been presented in Figure 1.

Table 1 represents the gender-wise distribution of the number of participants in both Easy and Hard groups. 

**Table 1:** Gender-wise distribution of the number of participants in Hard and Easy groups

	Female	Male	Prefer Not to say	Total
Easy	160	155	10	325
Hard	174	120	6	300
Total	334	275	16	625

**Figure 2:**
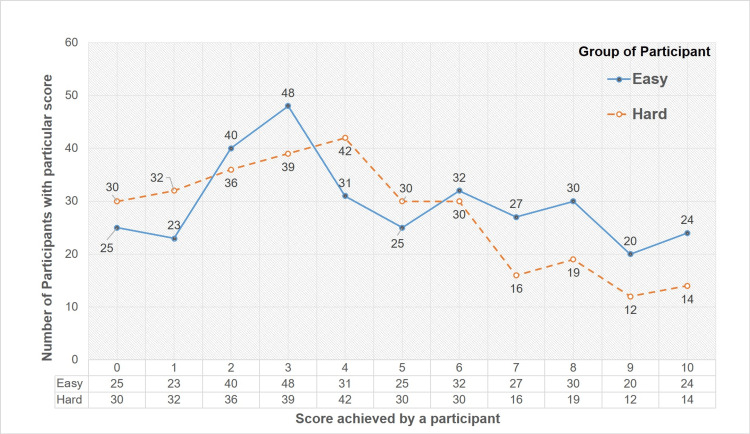
The frequency distribution of the participants' scores This chart shows the frequency distribution of the participants according to the scores achieved by them. The X-axis represents the respective score achieved by a participant and Y-axis shows the number of participants with that particular score. The line chart shows the trend in the number of participants over the range of scores. The solid line represents the Easy group and the dashed line represents the Hard group.

The frequency distribution of the participants according to the score achieved by them in both Easy and Hard groups is represented graphically in Figure 2. The line chart represents the trend of the number of participants at a particular score. The trend depicted by the graph reveals that a larger number of individuals achieved higher scores in the Easy group when compared to the Hard group. In contrast, a greater number of participants obtained lower scores in the Hard group when compared to the Easy group. This difference is further assessed by performing a t-test on the mean scores of participants in both groups.

Comparison between scores of Hard and Easy groups

The mean score of the participants who were primed to be in the Easy group was higher than those who were primed to be in the Hard group. The 325 participants to whom the questions were described as Easy (M = 4.73; SD = 2.98) demonstrated statistically significantly better scores (P two tail < 0.001)( t Stat = 2.704; t(623) = 1.96) compared to the 300 participants to whom the questions were described as Hard (M = 4.1; SD = 2.8). The result of the t-test performed between the mean score of the Easy and Hard groups is described in Table 2. 

**Table 2:** Results of the t-test performed between the mean score of Easy and Hard groups Both one-tailed and two-tailed p-value is <0.05 suggesting that the difference between the mean scores of the Easy and Hard groups is statistically significant with the mean score of the Easy group being significantly more than the mean score of the Hard group; thus, rejecting the null hypothesis that there is no significant difference between the scores of the Hard and Easy groups.

Group	Easy	Hard
Mean Score of the group	4.729	4.103
Variance	8.896	7.859
Number of Observations	325	300
Hypothesized Mean Difference	0	
df	623	
t Stat	2.704	
P(T<=t) one-tail	0.004	
t Critical one-tail	1.647	
P(T<=t) two-tail	0.007	
t Critical two-tail	1.964	

Comparison between scores of females and males

Between males and females, female participants had an overall higher mean score compared to that male participants. The 334 participants who were females (M = 4.77, SD =2.95) demonstrated statistically significantly better scores (P two-tailed < 0.001). (t Stat = 3.02; t Critical = 1.96) compared to the 275 participants who were males (M = 4.05, SD = 2.83). The result of the t-test performed between the mean score of females and males is described in Table 3. 

**Table 3:** Results of the t-test performed between the mean scores of females and males Both one-tailed and two-tailed p-value is <0.05 suggesting that the difference between the mean scores of females and males is statistically significant with the mean score of females being significantly more than the mean score of males.

Gender	Female	Male
Mean Score of the group	4.766	4.055
Variance	8.720	8.015
Number of Observations	334	275
Hypothesized Mean Difference	0	
df	607	
t Stat	3.029	
P(T<=t) one-tail	0.001	
t Critical one-tail	1.647	
P(T<=t) two-tail	0.003	
t Critical two-tail	1.964	

Stratifying participants based on their primed group

In both Hard and Easy groups, the statistically significant difference between males and females was still present upon stratifying participants based on their primed groups. In the Hard group, the 174 female participants (M = 4.40, SD = 2.8 ) demonstrated statistically significantly better scores (P two tail = 0.0436) ( t Stat = 2.44; t Critical = 1.96) compared to the 120 male participants (M = 3.67, SD =2.71). In the Easy group, the 160 female participants (M = 5.16, SD = 3.03) compared to the 155 male participants (M = 4.35, SD =2.89) demonstrated statistically significantly better scores (P two tail = 0.016). (t Stat = 2.42; t Critical = 1.96)

Stratifying participants based on their gender

In both females and males; the statistically significant difference between Hard and Easy groups was still present upon stratifying participants based on their gender. In males, the 155 participants to whom the questions were described as Easy (M = 4.35, SD = 2.89) demonstrated statistically significantly better scores (P two tail = 0.0436). (t Stat = 2.027; t Critical = 1.96) compared to the 120 participants to whom the questions were described as Hard (M = 3.67, SD = 2.7). In females, the 160 participants to whom the questions were described as Easy (M = 5.16, SD =3.03 ) demonstrated statistically significantly better scores (P two tail = 0.019) ( t Stat = 2.359; t Critical = 1.96) compared to the 174 participants to whom the questions were described as Hard (M = 4.4, SD = 2.84).

## Discussion

Priming has generally referred to the facilitative effects of some event or action on subsequent associated responses [10]. Within social psychology, this process has specifically come to be defined in terms of how such events or actions influence the activation of stored knowledge [4][11].

The results of our study support our alternate hypothesis, which was to have a more positive outcome for those who are positively primed, and a more negative outcome for the negatively primed, as the difference between the mean scores of the two groups was found to be statistically significant, with ones primed positively having an overall better performance than the ones primed negatively.

A previously conducted study found that participants primed to view the task as "easy" demonstrated a higher level of interest than those primed to view the task as "hard" [12]. Similar inferences can be made from the results of our study as those primed to view the question as "easy" scored higher compared to those primed to view the question as "hard," possibly indicating a higher level of interest in the former group.

A similar study suggested that the ones who perceived the task of completing three anagram worksheets to be hard had worse performance compared to ones who perceived it to be easy [13], which conforms to our study. Another study suggested that the description of the difficulty level as hard caused intimidation and a subsequent reduction of effort and/or misuse of time in the form of looking for difficulty where it is nonexistent [14]. However, a similar study also suggested that the ones instructed for the task being hard had better performance than the ones instructed that the task being easy [15], which is in contradiction to our study’s results. Some previously conducted studies suggested that the effects of priming on performance may be mediated by factors such as higher anxiety and related bodily responses such as raised heart rate related to problem-solving [16,17], motivation/effort [18,19], and self-efficacy [20].

As stated in the book *Learning and Memory: A Comprehensive Reference, 2008*’, “the priming effect (i.e., improvement in performance) is largest when the repeated stimulus is identical to the initial stimulus (prime)” [8]. In our study, we use a similar method to induce the priming effect, i.e., by repeatedly stating that the question is Easy/Hard, according to the participant’s assigned group. A study found that repeated exposure to a stimulus, which serves as a priming factor for the event, influences the outcome of the study [7]. In our study, the perceived fact about the questions being "hard" or "easy" would have affected the response of an individual to the question (here the stimulus being the difficulty of the question).

The results of this study demonstrate that the difference between the mean scores of female and male groups was found to be statistically significant, with the mean scores of female participants being higher. This could be attributed to the fact that men usually have higher impulsivity and carelessness while solving problems compared to women [21]. However, another study contradicted the aforementioned findings, saying that male subjects tend to have higher critical thinking and problem-solving abilities [22]. A similar study comparing various cognitive abilities between the two genders stated that the female subjects had higher verbal cognitive abilities than the male subjects. The Ditloid puzzles used in this study can also be considered a verbal cognitive test since it tests the ability to recall various words and form them into complete sentences and may explain better performance in the female group [23,24]. Another similar study concluded that female subjects had performed at a higher level in a verbal episodic memory task when compared to male subjects [25].

The difference between mean scores of the Hard and Easy groups in both males and females was similar from which it can be concluded that gender was not a mediating factor in the effects of priming.

The limitations of this study mainly arise from the use of an online form-based questionnaire as a research modality, which posed difficulties in ensuring fair means of answering. Another limitation pertains to the potential for more effective priming if the subjects are physically present during the test.

## Conclusions

Our study reveals that priming individuals to perceive a task as easy can result in better performance outcomes compared to priming them to perceive it as difficult. This highlights the crucial role of priming in enhancing performance. We also found gender differences in verbal cognitive abilities, with females performing better than males.

These findings can have significant implications for education and the workplace, emphasizing the need for individualized approaches to optimize performance outcomes. By considering factors such as priming and individual differences in cognitive abilities, we can better understand and enhance performance outcomes in various contexts.
